# Minor Stroke and Transient Ischemic Attack: Research and Practice

**DOI:** 10.3389/fneur.2016.00086

**Published:** 2016-06-10

**Authors:** Aleksandra Yakhkind, Ryan A. McTaggart, Mahesh V. Jayaraman, Matthew S. Siket, Brian Silver, Shadi Yaghi

**Affiliations:** ^1^Department of Neurology, The Warren Alpert Medical School of Brown University, Providence, RI, USA; ^2^Department of Diagnostic Imaging, The Warren Alpert Medical School of Brown University, Providence, RI, USA; ^3^Department of Neurosurgery, The Warren Alpert Medical School of Brown University, Providence, RI, USA; ^4^Department of Emergency Medicine, The Warren Alpert Medical School of Brown University, Providence, RI, USA

**Keywords:** minor stroke, transient ischemic attack, minor stroke outcome, treatment, recurrent stroke risk, stroke prevention

## Abstract

A majority of patients with ischemic stroke present with mild deficits for which aggressive management is not often pursued. Comprehensive work-up and appropriate intervention for minor strokes and transient ischemic attacks (TIAs) point toward better patient outcomes, lower costs, and fewer cases of disability. Imaging is a key modality to guide treatment and predict stroke recurrence. Patients with large vessel occlusions have been found to suffer worse outcomes and could benefit from intervention. Whether intravenous thrombolytic therapy decreases disability in minor stroke patients and whether acute endovascular intervention improves functional outcomes in patients with minor stroke and known large vessel occlusion remain controversial. Studies are ongoing to determine ideal antiplatelet therapy for stroke and TIA, while ongoing statin therapy, surgical management for patients with carotid stenosis, and anticoagulation for patients with atrial fibrillation have all been proven to decrease the rate of stroke recurrence and improve outcomes. This review summarizes the current evidence and discusses the standard of care for patients with minor stroke and TIA.

## Background

In population-based studies, approximately two-thirds of ischemic stroke patients have mild deficits (1, 2). Minor stroke is generally defined as an National Institute of Health Stroke Scale (NIHSS) of 5 or less, which takes into account certain deficits but not the fact that some can have a more profound impact on quality of life than others. Hence, the scale does not linearly correlate deficit and disability. While studies suggest using an NIHSS of 3 or less to define minor stroke (3, 4), real-world definitions of non-disabling deficits are largely dependent on clinical judgment, which has been shown to vary widely among physicians (5). Transient ischemic attack (TIA) has a more widely accepted definition that includes focal neurological symptoms lasting for <24 h without the presence of infarction on diffusion-weighted imaging (6).

In clinical practice, both minor stroke and TIA patients undergo similar diagnostic evaluations. Due to the relatively high early risk of stroke recurrence in both groups and disability in minor stroke patients, key decisions in the management of minor strokes and TIA can have significant impacts on clinical outcomes, quality of life, and cost of care. In this review, we summarize the current research on minor stroke and TIA, and highlight key points in acute treatment and secondary stroke prevention strategies.

## Acute Treatment

### Thrombolytic Therapy

Thrombolytic therapy with intravenous recombinant tissue plasminogen activator (IV rtPA) is an important treatment for patients with acute ischemic stroke (7). Patients with minor deficits are often excluded from such treatment even though they demonstrate a high rate of suboptimal functional outcome. While retrospective studies show no benefit in 3-month outcome between thrombolysed and non-thrombolysed patients with mild deficits (8, 9), these studies are subject to selection bias in favor of treating patients with disabling versus non-disabling deficits. Recent evidence from a stroke registry supports the use of IV rtPA compared with routine medical management in patients with mild deficits (10). In addition, a *post hoc* analysis of the International Stroke Trial-3 (IST-3) found that patients with mild deficits who were treated IV rtPA compared to placebo had a favorable shift in the Oxford Handicap Scale distribution (adjusted odds ratio, 2.38; 95% confidence interval, 1.17–4.85). The most feared complication of IV rtPA is symptomatic intracerebral hemorrhage (sICH), which is seen in up to 2% of patients with minor stroke (11–13). Due to the fear of hemorrhagic complications, physicians tend to offer IV rtPA to patients who they consider to have a disabling deficit, a highly subjective clinical judgment. The subjectivity of this approach highlights the need for prospective cohorts to better understand the natural history and predictors of long-term functional and cognitive outcomes in patients with minor stroke, taking into account the type of deficit, the patient, and potential for stroke recovery. Two randomized clinical trials comparing IV rtPA versus placebo in patients with minor non-disabling deficits are underway (14, 15).

### Acute Endovascular Therapy

Several clinical trials recently showed that the addition of mechanical thrombectomy to best medical treatment in patients with acute ischemic stroke and evidence of a large artery occlusion resulted in significant improvement in long-term functional outcomes (16). Most of these studies excluded patients with minor stroke leading to variability in the use of mechanical thrombectomy for patients with acute large vessel occlusion and transient or minor deficits. Large vessel occlusion is an important and consistent predictor of neurological deterioration and disability in patients with minor stroke (17, 18). Excluding these patients from endovascular treatment may lead to a sevenfold increased risk of long-term disabling deficits and up to 50% of patients being functionally disabled at 3 months (17, 18). Recent AHA/ASA guidelines suggest that it is reasonable to consider endovascular treatment for patients with an NIHSS score <6 and evidence of large vessel occlusion. However, clinical trials are needed to prove the efficacy of endovascular treatment in this patient population (19).

## Risk of Recurrent Stroke

The risk of recurrent stroke in patients with minor stroke and TIA is 10–13% at 90 days, with approximately half of events occurring in the first 2 days (20, 21). Multiple scores have been used to predict the early risk of stroke after a TIA or minor stroke, including the ABCD2 score (22, 23) and the ABCD3-I (24) score that include neurovascular and MR imaging (Table 1). Studies have shown imaging-supplemented scores to be superior to clinical scores alone in predicting stroke recurrence in patients with minor stroke or TIA (25, 26). In a meta-analysis of 29 studies and over 130,000 patients, the ABCD2 score lacked reliability in predicting early recurrent stroke risk and in identifying patients with symptomatic large artery atherosclerosis (27), an important prognosticator of early stroke recurrence (25, 28, 29). In fact, a recent multi-center study showed that in patients with minor stroke or TIA, the risk of early stroke recurrence or neurological deterioration was only up to 2% in the absence an infarction on neuroimaging or large artery disease stroke subtype and approximately 30% in those with large artery disease stroke subtype who have an infarction on neuroimaging. In this study, the ABCD2 score was not a predictor of stroke recurrence (30). Another study showed that the addition of perfusion imaging to parenchymal and vascular imaging in patients with minor stroke TIA improved prediction of recurrent cerebrovascular events (31).

**Table 1:** **Clinical stroke risk recurrence scores (23)**.

Score	ABCD2	ABCD3	ABCD3-I
**Components:**			
(1)	Age > 60 years	Age > 60 years	Age > 60 years
(1)	BP ≥ 140/90 mmHg Clinical features:	BP ≥ 140/90 mmHg Clinical features:	BP ≥ 140/90 mmHg Clinical features:
(1)	– Speech impaired w/o weakness	– Speech impaired w/o weakness	– Speech impaired w/o weakness
(1)	– Unilateral weakness	– Unilateral weakness	– Unilateral weakness
(1)	– Duration 10–59 min	– Duration 10–59 min	– Duration 10–59 min
(2)	– ≥60 min	– ≥60 min	– ≥60 min
(1)	– Diabetes Mellitus	– Diabetes Mellitus	– Diabetes Mellitus
(2)		– Dual TIA within 7 days	– Dual TIA within 7 days
(2)			– Intra and extracranial imaging with ≥50% stenosis of ipsilateral large artery
**Approximate stroke incidence (%) in high-risk patients after**	**(Score 6–7)**	**(Score 6–9)**	**(Score 8–13)**
7 days	10	9	14
90 days	12	12	15
3 years	28	28	30

## Secondary Stroke Prevention

### Acute Antiplatelet Agents

Due to the relatively high early risk of recurrent stroke in patients with minor stroke or TIA as compared to those with larger stroke severity, secondary stroke prevention is one of the most important steps in improving outcome in this patient population. Secondary prevention strategies include the use of antiplatelet agents, statins, and aggressive risk factor modification (32). Aggressive therapies targeting platelet inhibition in the absence of indications for anticoagulation is of paramount importance. Aspirin when administered within 48 h of an ischemic stroke has been shown to be effective in reducing the 2-week risk of stroke (2.8–3.3 versus 3.9%) (33, 34). The *Clopidogrel with Aspirin in Acute Minor Stroke or Transient Ischemic Attack* (CHANCE) trial, which included patients with TIA and minor stroke (NIHSS score ≤3) and excluded patients with isolated sensory symptoms, isolated dizziness, or isolated visual changes found an advantage of short-term dual antiplatelet therapy (aspirin plus clopidogrel) over aspirin monotherapy in the occurrence of secondary stroke (20). An important caveat about the CHANCE trial is that less than half of patients received a lipid-lowering drug and only about one-third received antihypertensive treatment, which is a very different management style in North America. In addition, the event rates, which were close to 10% in both arms far exceed the current rate of secondary stroke in North America – estimated at <5%. The ongoing Platelet-Oriented Inhibition in New TIA and Minor Ischemic Stroke Trial (POINT) is assessing a similar population in North America with more robust use of statins and antihypertensive agents to see whether the results observed in CHANCE will be replicated. Short-term dual antiplatelet therapy has been used for secondary stroke prevention in patients with symptomatic intracranial atherosclerotic disease (35), symptomatic carotid disease to reduce the number of micro-embolic signals (36), and in those with stuttering lacunar stroke (37, 38) with a potential reduction of stroke risk. While the bleeding risk of long-term dual antiplatelet therapy is higher than that of aspirin alone (39), there is evidence to support the safety of such treatment when used on a short-term basis when the risk of recurrent stroke is highest without an increase in ICH (20). Furthermore, the short-term use of ticagrelor, another antiplatelet agent used for acute coronary syndrome, was compared with aspirin in patients with TIA and minor stroke in the SOCRATES trial (Acute Stroke Or Transient IsChaemic Attack TReated With Aspirin or Ticagrelor and Patient OutcomES). Results of this trial showed no significant difference in recurrent events between the two groups.

### Statin Therapy

In addition to their effect on lowering cholesterol, statins have neuroprotective and anti-inflammatory properties that enhance endothelial function and promote blood flow (40). A small randomized study (41) and a larger retrospective study (42) showed that patients who were continued on a statin acutely after a stroke had a better outcome than those in whom the statin was withdrawn. Therefore, in patients with minor stroke or TIA, early continuation of statin therapy may improve both short-term and long-term stroke prevention strategies.

### Carotid Revascularization

Evaluation for carotid stenosis is a key step in the diagnostic evaluation of patients with TIA and minor stroke. In patients with this disease, surgical management in addition to best medical therapy is superior to best medical therapy alone, particularly in those with >70% symptomatic stenosis (43). While medical treatment for stroke prevention has improved, carotid endarterectomy or carotid artery stenting still remain an integral part of the treatment for patients with severe symptomatic carotid stenosis. Since the risk of recurrent stroke in such patients is highest in the first 2 weeks after their initial event (44), the AHA/ASA recommends urgent surgery within 2 weeks of symptoms (32).

### Atrial Fibrillation Detection

Identifying atrial fibrillation is important in secondary stroke prevention as anticoagulation is superior to antiplatelet agents in reducing the stroke risk of patients with atrial fibrillation. Electrocardiogram and inpatient cardiac telemetry should be part of diagnostic evaluation of patients admitted with ischemic stroke (32). Longer cardiac monitoring via mobile continuous outpatient telemetry (MCOT) or implantable loop records may be indicated in patients with cryptogenic stroke to increase the yield of detecting paroxysmal atrial fibrillation (45). Since the detection rates are higher with MCOT or loop recorders when compared to inpatient telemetry, patients with mild deficits do not need to be admitted solely for inpatient telemetry. Detection rates of paroxysmal atrial fibrillation using various cardiac monitoring are shown in Table 2.

**Table 2:** **Detection rates of paroxysmal atrial fibrillation using various cardiac monitoring strategies (42)**.

Type of monitoring	Setting	Duration	Rate of detection of atrial fibrillation (%)
Admission ECG	Inpatient	N/A	2.7
Inpatient continuous telemetry	Inpatient	24–48 h	5.5
Holter monitor	Outpatient	24–48 h	3.2–6.4
Mobile continuous outpatient telemetry	Outpatient	21–28 days	16–25
Implantable loop recorders	Outpatient	6 months	9
		36 months	30

## Triaging and Disposition

Any acute, symptomatic stroke-like symptom (transient or not) should be considered a medical emergency. Interventions should be limited at this point to streamline the acquisition of a non-contrast CT scan of the brain to exclude the presence of any intracranial hemorrhage. Patients in whom an acute large vessel occlusion of the anterior or posterior circulation is suspected should be considered for upfront CT angiographic imaging as well. An exception is made for patients who experienced entirely fleeting symptoms and have returned to their neurologic baseline. In these patients, a CT scan can be forgone in lieu of a diffusion-weighted MRI provided hospital resources allow for urgent imaging acquisition (within 24 h) and the patient has no contraindications to MRI. As recommended by the AHA/ASA, all patients with TIA should undergo brain and cervico-cephalic vascular imaging routinely as part of their initial evaluation (7).

A detailed neurological exam that includes, but is not limited, to an NIHSS should be completed in all cases. The provider should remain vigilant of subtle neurological dysfunction, as patients may report symptom resolution, but still exhibit focal abnormalities. This occurred in 26% of patients referred to a same-day TIA clinic when examined by a neurologist in one study (46).

Determining which TIA patients require inpatient hospitalization is largely a factor of institutional resources and the ease of access for patients to receive follow-up care. As such, significant practice variability exists between providers and institutions and this has been a source of frequent debate. On the whole, somewhere between 53 and 91% of all TIA patients presenting to U.S. emergency departments (EDs) are admitted (47–53). The focus should be on determining the underlying etiology of the ischemic event and optimizing individual secondary prevention strategies. Large artery atherosclerosis can be effectively ruled out with brain imaging and vascular imaging of the head and neck (CTA, MRA, or carotid Duplex and transcranial Doppler ultrasound). Cardioembolic etiologies should be explored with electrocardiogram and in-house telemetry, followed by echocardiography and prolonged holter monitoring in selected patients. The suspected etiology of the ischemic event should be classified into the best of the provider’s ability at the time of the initial encounter. ED wait times and limited emergency neurology expertise availability are common drivers for hospitalization. Another determinant of hospitalization is the presence of disabling deficits in gait or swallowing requiring inpatient physical and speech therapy evaluations.

Rapid access TIA clinics and ED observation units provide efficient and cost-effective alternatives to hospitalization for many patients. Observation units, typically located within or near the hospital EDs, provide expedited and protocol-driven care and have a proven track record of safety with TIA (54–57). Outpatient clinics, such as the French SOS-TIA and British EXPRESS models, have successfully implemented 24/7 urgent outpatient follow-up for TIA, while reducing 90-day stroke recurrence by 80% (58–60). As stroke systems of care are increasingly regionalized and hospitals become increasingly incentivized to optimize cost-effective care and decrease inpatient length of stay, these models will likely be increasingly utilized.

Risk stratification tools, such as the ABCD2 score, have been implemented in some systems to triage the urgency of follow-up or determine in whom hospitalization is advised. Current NICE criteria and AHA/ASA guidelines both support this practice, which was found to be safe in a recent Australian study (6, 7, 53, 61). However, as discussed in the preceding section, clinical risk stratification tools are rather imperfect and the more robust-imaging enhanced tools include definitive (and often inpatient) advanced imaging modalities. While clinical risk prediction may be used to augment one’s gestalt of a patient’s short-term recurrent stroke risk, it should not be used as the sole determinant of disposition. Adopting a frontloaded, etiology-focused work-up that optimizes individual secondary prevention strategies is likely to be the best approach, regardless of location (hospital, clinic, or observation unit) (62).

## Outcome

Approximately 30% of patients with minor strokes have poor functional outcomes [defined as modified Rankin scale (mRS) score of 2–6] at 90 days (3, 63). Furthermore, nearly one-third of patients admitted to the hospital for a mild stroke are not discharged home (64, 65) and cannot walk independently at discharge (64). A consistent predictor of outcome in patients with minor deficits is evidence of large vessel occlusion on imaging (17, 18). However, absence of large vessel occlusion is also associated with substantial long-term disability (17, 18). Studies exploring the type of deficits as predictors of outcome in patients with minor stroke yield mixed results. One study showed that the types of deficits do not predict outcome in minor stroke (66). Another study showed that certain items not included in the NIHSS score, such as distal hand weakness and gait disorder, increase the likelihood of a poor functional outcome after a minor stroke (67). Other items of the NIHSS that have been associated with poor outcome in studies include language deficits (67), neglect (68), and leg weakness (68). The NIHSS score and outcome measures assessed in prior studies, such as mRS can underestimate cognitive deficits (69), particularly visuospatial or executive dysfunction (70), and post-stroke fatigue (71) that can occur after a minor stroke. These deficits may cause long-term disability after a mild stroke. Therefore, capturing more sensitive impairment measures that include gait speed, simplified neuropsychological assessments, and examination of distal hand strength and dexterity may aid in making treatment decisions in patients with otherwise mild deficits. Patient-related factors, such as baseline functional status, hobbies, and occupation, may also help treating physicians decide the degree to which the nature of deficits may affect patients’ functional outcome.

## Conclusion

A comprehensive approach to the treatment and triaging of patients with minor stroke or TIA may lead to decreased long-term functional deficits in this patient population and still be cost-effective. Randomized trials exploring several treatments and outcome predictors in patients with TIA and minor stroke are underway and aim to advance management and reduce the degree of functional disability.

## Author Contributions

AY: drafting of manuscript, critical revisions, and final approval. RM: critical revisions and final approval. MJ: critical revisions and final approval. MS: drafting of manuscript, critical revisions, and final approval. BS: critical revisions and final approval. SY: drafting of manuscript, critical revisions, and final approval.

## Conflict of Interest Statement

The authors declare that the research was conducted in the absence of any commercial or financial relationships that could be construed as a potential conflict of interest.
